# Empirical analysis comparing the tele-objective structured clinical examination and the in-person assessment in Australia

**DOI:** 10.3352/jeehp.2021.18.23

**Published:** 2021-09-23

**Authors:** Jonathan Zachary Felthun, Silas Taylor, Boaz Shulruf, Digby Wigram Allen

**Affiliations:** 1 School of Medicine, The University of New South Wales, Kensington, Australia; 2 Office of Medical Education, University of New South Wales, Sydney, Australia; 3 Centre for Medical and Health Sciences Education, Faculty of Medical and Health Sciences, University of Auckland, Auckland, New Zealand; Hallym University, Korea

**Keywords:** Educational technology, Objective structured clinical examination, Online assessment, Remote medicine, Telemedicine, Australia

## Abstract

**Purpose:**

It aimed to compare the use of the tele-objective structured clinical examination (teleOSCE) with in-person assessment in high-stakes clinical examination so as to determine the impact of the teleOSCE on the assessment undertaken. Discussion follows regarding what skills and domains can effectively be assessed in a teleOSCE.

**Methods:**

This study is a retrospective observational analysis. It compares the results achieved by final year medical students in their clinical examination, assessed using the teleOSCE in 2020 (n=285), with those who were examined using the traditional in-person format in 2019 (n=280). The study was undertaken at the University of New South Wales, Australia.

**Results:**

In the domain of physical examination, students in 2020 scored 0.277 points higher than those in 2019 (mean difference=–0.277, P<0.001, effect size=0.332). Across all other domains, there was no significant difference in mean scores between 2019 and 2020.

**Conclusion:**

The teleOSCE does not negatively impact assessment in clinical examination in all domains except physical examination. If the teleOSCE is the future of clinical skills examination, assessment of physical examination will require concomitant workplace-based assessment.

## Introduction

### Background/rationale

The coronavirus disease 2019 (COVID-19) pandemic has forced healthcare education across the globe to adapt to the unique and challenging scenario of facilitating performance-based examinations under strict social distancing requirements. Many “pivoted” to online platforms with minimal guidance from the literature, to implement these assessments [1-3]. Theoretically, the principles of objectivity and structure that underpin the exam, should be replicable in the online format and consistently achieved when broadcasting the same patients to all students in different locations. However, assessment of the performance of skills has evolved beyond the early design of the objective structured clinical examination (OSCE), becoming more intricate and nuanced, and implemented in both academic and employment contexts [4,5]. The incorporation of subjectivity through the re-popularized global rating scales has permitted the assessment of practices that are difficult to quantify with a binary marking scheme, such as building rapport or demonstrating empathy [6,7]. The implementation of an online format signals a further departure from its historical design. Recent efforts to deliver the tele-objective structured clinical examination (teleOSCE) and online OSCE orientation have been acceptable to students and examiners respectively [8,9]. A systematic review summarized the various attempts in the literature to incorporate an online element and discovered that while there is generally good validity and reliability, there is a need for systemic research to guide the ideal teleOSCE format [10]. As yet, no study has attempted to define and discuss the impact on the assessment outcome of separating students, patients, and assessors into different locations, and executing examinations online. This is of paramount concern in the context of current (and ongoing) social distancing requirements, whilst also providing useful insights into improving the accessibility of examinations to relevant stakeholders.

The challenging year of 2020 has offered an unprecedented and valuable opportunity for data collection on the online assessment process. The University of New South Wales in Sydney, Australia is one of many medical schools that was forced to use an online “electronic” OSCE (teleOSCE) with minimal guidance. Final year students underwent their summative OSCE assessments using this online format to comply with COVID-19 restrictions.

### Objectives

The following study aims to establish whether the use of the teleOSCE has impacted assessment outcomes. Ultimately, the study aims to facilitate discussion on what skills and domains can effectively be assessed in a teleOSCE to guide future development and use of online clinical examinations.

## Methods

### Ethics statement

The research was conducted under the ethics approval granted by the Human Research Ethics Committee of the University of New South Wales (reference no., HC15421, HREAPG: Health, Medical, Community and Social). Complying with the ethics approval we used administrative assessment data held by the University of New South Wales which required no consent from participants.

### Study design

This study is an observational study which compares the results achieved by the students examined in 2020 via the teleOSCE, with those who were examined under the traditional OSCE format in 2019.

### Setting

In-person clinical OSCEs have been run for many years and staff are familiar with the requirements for implementation. Conversion of this to an online format required several steps. The prime amongst these was the technology aspect (for all participants) which was entirely novel in this setting. Thereafter, adaptation of the station was required, i.e., to suit an online format. In conjunction, personnel and procedural changes were required, before various stages of testing. The clinical stations are described below, with the technological, personnel, procedural, testing, and training aspects described in Supplement 1.

The traditional in-person OSCE, as run in 2019, consisted of 9 stations from 7 disciplines (medicine, surgery, primary care, emergency medicine, psychiatry, pediatrics, and obstetrics and gynecology) of 12 minutes duration. For each station students are provided with a short summary of a clinical scenario, from which they are expected to take a brief targeted history, and conduct a physical examination, before attempting to diagnose the patient, suggest relevant investigations and management, and answer questions from the examiner. Each station is preceded by 2 minutes reading time and followed by a short period to allow transition to the subsequent station. Within stations students are assessed on their clinical skills in relation to the specific station content, but also on generic aspects such as communication and physical examination skills. To ensure comparability between the teleOSCE, and the in-person OSCE, core features from the latter were maintained in the online version wherever possible. For the most part, this was relatively straightforward since initiation of the consultation with the patient, collection of the history, interpretation of clinical information and summarization of the case by the student could all be easily achieved in the online format (Supplement 2). However, for the physical examination and/or procedural skills components, these aspects needed to be adapted into an activity which could be completed and assessed online. Here the task now required the student to describe the approach to and process of examining the patient in whatever way they felt the station question and information elicited suggested (Table 1). These changes also necessitated modification of the assessment rubrics. To this end, each adapted OSCE station was reviewed by staff familiar with clinical assessment and with recommendations made to station authors should further changes be required to suit the online format.

### Participants

For the teleOSCE in 2020, 285 senior students participated in the assessment; while, for the traditional in-person format evaluation of OSCE in 2019, 280 senior students participated. All target students participated in the examinations.

### Variables

Scores of the teleOSCE examination and that of the traditional in-person OSCE.

### Data source/measurement

The results of teleOSCE and traditional in-person OSCE for 2 years. The data used in this analysis were the raw scores given by the examiners before modifications made by the post examination standard setting process implemented by the medicine program [11,12]. This process was essential to allow the analysis focusing on examiners’ assessment outcomes rather than overall outcomes for students which could be impacted by the standard setting method.

### Bias

There was no sampling bias because all target students participated in the OSCE and all data was included in the study.

### Study size

The sample size of this study was determined by the availability of participants, all of which were included in the study (N=565; combined cohorts of 280 and 285 each). Because the study used all available data and the study had no impact on the participants it was unnecessary to limit the number of study participants nor undertake a priori sample size and power calculation [13]. The results do present the effect size which is the appropriate measure to estimate the meaning of the differences between the 2 cohorts [14]. No post-hoc sample size and power calculation were undertaken because these are not statistically appropriate [15].

### Statistical methods

Independent t-test analysis was used to compare the mean scores of student assessment results between 2019 (in-person OSCE) and 2020 (teleOSCE). The comparison was made by disciplines (all assessment domains combined) and separately by domains (all disciplines combined). This analysis was chosen to identify the impact of the assessment mode on different medical disciplines as well as assessment domains, respectively. Bonferroni correction was undertaken to correct for alpha inflation.

## Results

### Descriptive data of participants

In 2019, 280 students undertook the in-person examinations, while in 2020, there were 285 examinees in the teleOSCE. All students completed the entirety of their examinations, and all of their unidentified raw scores were eligible for use in this study. Demographic data was not available for inclusion in this study.

### Main results

In the domain of physical examination, students in 2020 scored 0.277 points higher than those in 2019 (mean difference=–0.277, P<0.001, effect size=0.332). Across all other domains, there was no significant difference in mean scores between 2019 and 2020. These results are illustrated in Table 2 and Dataset 1.

When analyzing the results by discipline, compared to the 2019 (in-person) students the 2020 (teleOSCE) students scored 0.216 points higher in medicine (mean difference=–0.216, P<0.05, effect size=0.257) and 0.390 points higher in emergency medicine (mean difference=–0.390, P<0.01, effect size=0.363) respectively. Across all other disciplines, there was no significant difference between the mean scores in 2019 compared to 2020. These results are depicted in Table 3 and Dataset 1.

## Discussion

### Key results

The cohorts in this study were of comparable size (280 versus 285) and no significant difference were seen in the outcomes of almost all domains (communication, clinical history and background, medical knowledge, interpretation, diagnosis, and management) and disciplines. This suggests that the change to an online platform did not influence the performance of students, or the information available to assessors in these areas. However, significant differences are noted in the disciplines of medicine and emergency medicine, and the domain of physical examination.

### Interpretation

The method of performance assessment utilized in 2019 has been in practice at the University of New South Wales for many years and minor adaptations in methodology have previously been well-described in the literature [11]. There are several potential explanations for the higher mean scores in medicine (mean difference=–0.216, P<0.05) and emergency (mean difference=–0.390, P<0.01) between the 2 cohorts. Most notably, the in-person OSCE at University of the New South Wales was the product of successive annual reviews and moderation informed by local data and educational research. Conversely, the teleOSCE was the culmination of 6 months of targeted design and innovation with a paucity of guidance from the literature. As such, this may have limited the case or task complexity incorporated into several of the teleOSCE stations and this could have contributed to the improvement in assessment outcomes observed in the medicine and emergency medicine stations. For instance, the adaption of multiple procedural tasks performed in the emergency medicine station required greater consideration than in, for example, a psychiatry station, in which the patient history and mental state examination translated quite straightforwardly to the teleOSCE format. Additionally, there are multiple established examiner-specific variables that could have further influenced the difference observed. For instance, examiner training has been shown to decrease marking variation [16]. Examiner expertise may be inversely correlated with generic scores in communication, while seniority may be associated with lower scores in general [17]. With the rapid development and implementation of the teleOSCE, expertise, training and issues around seniority of examiners were less pertinent in a brand-new platform. This foreseeably could impact disciplines unevenly, given the varied suitability of each discipline to the teleOSCE platform (e.g., emergency medicine versus psychiatry).

Exploring adjustments made to the method of examination provides insights into why the physical examination domain results may have changed (Table 1). In both the in-person and online formats, students are required to plan the physical examination they would like to undertake and in the teleOSCE this plan had to be clearly articulated to the examiner. In the latter, the physical findings were provided upon specific student request. In contrast, students undertaking the in-person iteration were required to gather this information using their physical examination skills. In both formats, students then used their clinical reasoning to construct an appropriate list of differential diagnoses. As such, poor performance in physical examination may have hampered information gathering for students undertaking the in-person assessment, whereas the online format negated this deficiency by affording the student the necessary clinical information on request to formulate their diagnosis.

Additionally, the information available to the examiner is less in the teleOSCE. Previously, the in-person format permitted an examiner a single observation of the student, with multiple facets, including witnessing the students carry out the steps of an examination. By removing this specific data point, examiners simply have less information with which to make a judgement, a well-established hindrance to validity [18]. Observation of physical examination has been the cornerstone of performance examination in the OSCE format for decades, and this result challenges the value of assessing physical examination in the teleOSCE in its current format. Amending the configuration of the teleOSCE to permit the appropriate performance of physical examination skills represents a potential alternative. For instance, this could be achieved by placing the student together with the patient in a single room, leaving only the assessor in a remote location. This has been studied previously, with positive results in terms of acceptability of participants and information gleaned by examiners [19]. However, with the physical distancing requirements in place during the COVID-19 pandemic, this would not have been achievable in this study. Moreover, this method negates the improved accessibility to patients, a key benefit of the online platform. Instead, physical examination in the online platform could focus on signs that are transferrable through a screen. There is evidence through the applications of telehealth that physical examinations which rely heavily on observation such as dermatology are highly suited to this purpose [20]. Indeed an examination should represent the context it aims to simulate [21], and the teleOSCE represents the challenges of the telehealth consultation very well. It is easy to see its utility in training future clinicians for whom telehealth may become far more prevalent. But for its current purpose, that is assessing final year medical students across the various domains, there remains no current solution for a fully representative sample of physical examination skills in the teleOSCE, as the in-person format may have done.

Looking more broadly at performance examination, the observation of the performance of a single skill in a single environment during an assessment has poor generalizability due to case specificity [16]. For instance, if a student can appropriately listen to the precordium on a young fit simulated patient, they may not be able to reproduce the same performance in an unwell, overweight and/or elderly patient. Undeniably the only way to approach validity in assessment of a single target domain such as competence in physical examination, requires a multitude of observations in varied contexts [18]. To navigate the shortcomings of the teleOSCE in judging physical examination, other components of a multimodal assessment method will need to be further emphasized. We suggest that structured and cyclical clinical workplace-based assessment of physical examinations are highly suited to this purpose. Repeating observed clinical assessments and utilizing a structured feedback system (e.g., mini-clinical evaluation exercise) has been shown to provide students with a means to apply, evaluate and refine their clinical skills [22], whilst also allowing teleOSCEs to be utilized for assessment of the other domains of skills which we have shown to be unaffected by the online format.

### Limitations

Utilizing student marks to compare the impact of the in-person and teleOSCE has its limitations in that more than simply the student performance and the assessment modality will contribute to this result. Such confounders were not controlled for in this observational study. Furthermore, the in-person and teleOSCE assess different aspects of competence in the domain of physical examination and this may limit scope for comparison in this domain. Of note, the in-person OSCE requires a student to elicit findings by physical examination whereas the teleOSCE requires a student to describe this process (in stations requiring more information than can be gained by observation alone). The teleOSCE does impact assessment of physical examination competence; however, this may be due to the inherent constraints of the online format of assessment in this particular domain rather than solely ascribable to differences in student performance between 2019 and 2020.

### Conclusion

Research on teleOSCE is still in its infancy. Evaluating the method used by the University of New South Wales for performance examination in 2020 has demonstrated that the transition from the in-person performance assessment to a teleOSCE platform was very successful. Bar physical examination, comparing traditional in-person OSCE outcomes with those of the teleOSCE shows no significant changes across the various domains. As such, these online summative examinations can continue to play a role in the broader multimodal assessment system, providing valuable data to examiners when making an accurate judgement about a student’s competency in those domains. However, this study also revealed the need for careful consideration of the way we assess physical examination using teleOSCE. Students can no longer execute the actions of a physical examination on a patient, and as such, examiners have less information with which to make an assessment and this may account for an increase in scores in this domain. With the increased likelihood of utilizing teleOSCE more frequently in the future, we need to identify the physical examination skills that cannot be accurately assessed via teleOSCE and make sure these skills are assessed via workplace-based assessment tools.

## Figures and Tables

**Table 1.** Comparison of components of physical examination in the 2 different assessment formats

Method of the examination	In person	TeleOSCE
Planning a relevant physical examination	Formulated according to the station requirements, the information gleaned from history and observable features of the patient	Formulated according to the station requirements, the information gleaned from history and observable features of the patient
Performing the physical examination	Performed on the patient	Steps listed for examiner
Extracting clinical information	Interpreted from steps of the clinical examination	Clinical information is provided by the examiner to the student upon request
Formulating a diagnosis	Extrapolated from the clinical information garnered from previous steps	Extrapolated from the clinical information garnered from previous steps

TeleOSCE, tele-objective structured clinical examination.

**Table 2.** Comparison of 2019 and 2020 student results by different domains of the marking rubric

Domain	N		Mean		SD		SEM		t-value	Equal variance assumed	df	Sig.	Mean difference	95% CI	Effect size
	2019	2020	2019	2020	2019	2020	2019	2020							
Communication	280	285	7.618	7.569	0.621	0.571	0.0371	0.0338	0.971	Yes	563.00	0.332	0.049	-0.050 to 0.147	-0.082
Clinical history & background	280	285	7.090	7.147	0.866	0.691	0.0518	0.0409	-0.864	No	532.48	0.389	-0.057	-0.186 to 0.072	0.073
Medical knowledge	280	285	7.186	7.122	0.887	0.908	0.0530	0.0538	0.848	Yes	563.00	0.397	0.064	-0.084 to 0.212	-0.071
Physical examination	280	285	7.104	7.381	0.847	0.822	0.0506	0.0487	-3.947	Yes	563.00	<0.001^a)^	-0.277	-0.415 to -0.139	0.332
Interpretation	280	285	7.058	7.156	0.822	0.796	0.0491	0.0472	-1.445	Yes	563.00	0.149	0-.098	-0.232 to 0.035	0.121
Diagnosis & management	280	285	7.292	7.394	0.786	0.819	0.047	0.0485	-1.514	Yes	563.00	0.131	-0.102	-0.235 to 0.030	0.127

SD, standard deviation; SEM, standard error of the mean; df, degrees of freedom; Sig., significance; CI, confidence interval.

a) Significant at P<0.001 after correction for alpha inflation (Bonferroni correction).

**Table 3.** Comparison of 2019 and 2020 student results in the different sub-specialties

Discipline	N		Mean		SD		SEM		t-value	Equal variance assumed	df	Sig.	Mean difference	95% CI	Effect size
	2019	2020	2019	2020	2019	2020	2019	2020							
Medicine	280	285	7.275	7.492	0.768	0.921	0.0459	0.0546	-3.036	No	548.43	0.003^a)^	-0.216	-0.357 to -0.076	0.257
Surgery	280	285	7.376	7.354	0.851	0.934	0.0509	0.0553	0.298	Yes	563.00	0.766	0.022	-0.125 to 0.170	-0.025
Psychiatry	280	285	7.344	7.227	0.921	0.925	0.0550	0.0548	1.505	Yes	563.00	0.133	0.117	-0.036 to 0.269	-0.127
Emergency medicine	280	285	7.032	7.422	1.172	0.975	0.0701	0.0578	-4.304	Yes	563.00	<0.001^b)^	-0.390	-0.568 to -0.212	0.363
Obstetrics & gynecology	280	285	7.440	7.521	1.056	1.012	0.0631	0.0600	-0.934	Yes	563.00	0.351	-0.081	-0.252 to 0.090	0.078
Primary care	280	285	6.936	6.985	1.125	0.951	0.0672	0.0564	-0.565	No	544.63	0.572	-0.050	-0.222 to 0.123	0.047
Paediatrics	280	285	7.501	7.438	0.958	0.793	0.0573	0.0470	0.858	No	540.44	0.391	0.064	-0.082 to 0.209	-0.072

SD, standard deviation; SEM, standard error of the mean; df, degrees of freedom; Sig., significance; CI, confidence interval.

a) Significant at P<0.05 after correction for alpha inflation (Bonferroni correction).

b) Significant at P<0.01 after correction for alpha inflation (Bonferroni correction).

## Footnotes

**Data availability**
Data files are available from Harvard Dataverse: https://doi.org/10.7910/DVN/0XDXKS Dataset 1. Results of 2 objective structured clinical examination (OCSE) examinations of medical students in the University of New South Wales, Australia in 2019 and 2020.

## Supplementary materials

Data files are available from Harvard Dataverse: https://doi.org/10.7910/DVN/0XDXKS

Supplement 1. Technological, personnel, procedural, testing and training aspects of the tele-objective structured clinical examination.

Supplement 2. Video of tele-objective structured clinical examination (teleOSCE) interaction.

Supplement 3. Audio recording of the abstract.

## References

[1] Craig C, Kasana N, Modi A (2020). Virtual OSCE delivery: the way of the future?. Med Educ.

[2] Kakadia R, Chen E, Ohyama H (2020). Implementing an online OSCE during the COVID-19 pandemic. J Dent Educ.

[3] Rampton S, Tea C, Miesi K, Hamilton C (2020). PG30 How to do a remote OSCE successfully: converting face to face simulation into a virtual E-consult in the pandemic crisis. BMJ Simul Technol Enhanc Learn.

[4] Taylor S, Haywood M, Shulruf B (2019). Comparison of effect between simulated patient clinical skill training and student role play on objective structured clinical examination performance outcomes for medical students in Australia. J Educ Eval Health Prof.

[5] Nagoshi Y, Cooper LA, Meyer L, Cherabuddi K, Close J, Dow J, Markham MJ, Stalvey C (2019). Application of an objective structured clinical examination to evaluate and monitor intern’s proficiency of hand hygiene and personal protective equipment use in the United States. J Educ Eval Health Prof.

[6] Ilgen JS, Ma IW, Hatala R, Cook DA (2015). A systematic review of validity evidence for checklists versus global rating scales in simulation-based assessment. Med Educ.

[7] Daniels VJ, Pugh D (2018). Twelve tips for developing an OSCE that measures what you want. Med Teach.

[8] Elnaem MH, Akkawi ME, Nazar NI, Ab Rahman NS, Mohamed MH (2021). Malaysian pharmacy students’ perspectives on the virtual objective structured clinical examination during the coronavirus disease 2019 pandemic. J Educ Eval Health Prof.

[9] Khamisa K, Halman S, Desjardins I, Jean MS, Pugh D (2018). The implementation and evaluation of an e-Learning training module for objective structured clinical examination raters in Canada. J Educ Eval Health Prof.

[10] Felthun JZ, Taylor S, Shulruf B, Allen DW (2021). Assessment methods and the validity and reliability of measurement tools in online objective structured clinical examinations: a systematic scoping review. J Educ Eval Health Prof.

[11] Shulruf B, Damodaran A, Jones P, Kennedy S, Mangos G, O’Sullivan AJ, Rhee J, Taylor S, Velan G, Harris P (2018). Enhancing the defensibility of examiners’ marks in high stake OSCEs. BMC Med Educ.

[12] Klein Nulend R, Harris P, Shulruf B (2020). Predictive validity of a tool to resolve borderline grades in OSCEs. GMS J Med Educ.

[13] Kadam P, Bhalerao S (2010). Sample size calculation. Int J Ayurveda Res.

[14] Ellis PD (2010). The essential guide to effect sizes: statistical power, meta-analysis, and the interpretation of research results.

[15] Walters SJ (2009). Consultants’ forum: should post hoc sample size calculations be done?. Pharm Stat.

[16] Khan KZ, Ramachandran S, Gaunt K, Pushkar P (2013). The objective structured clinical examination (OSCE): AMEE guide no. 81. Part I: an historical and theoretical perspective. Med Teach.

[17] Chong L, Taylor S, Haywood M, Adelstein BA, Shulruf B (2018). Examiner seniority and experience are associated with bias when scoring communication, but not examination, skills in objective structured clinical examinations in Australia. J Educ Eval Health Prof.

[18] Schuwirth LW, van der Vleuten CP (2012). Programmatic assessment and Kane’s validity perspective. Med Educ.

[19] Chen TC, Lin MC, Chiang YC, Monrouxe L, Chien SJ (2019). Remote and onsite scoring of OSCEs using generalisability theory: a three-year cohort study. Med Teach.

[20] Bashshur RL, Shannon GW, Tejasvi T, Kvedar JC, Gates M (2015). The empirical foundations of teledermatology: a review of the research evidence. Telemed J E Health.

[21] Schuwirth LW, van der Vleuten CP (2011). General overview of the theories used in assessment: AMEE guide no. 57. Med Teach.

[22] Mortaz Hejri S, Jalili M, Masoomi R, Shirazi M, Nedjat S, Norcini J (2020). The utility of mini-clinical evaluation exercise in undergraduate and postgraduate medical education: a BEME review: BEME guide no. 59. Med Teach.

