# Supplementary material for: Empirical analysis comparing the tele-objective structured clinical examination and the in-person assessment in Australia
Source: J Educ Eval Health Prof. 2021 Sep 23;18:23. doi: 10.3352/jeehp.2021.18.23 (PMC8616724; doi:10.3352/jeehp.2021.18.23)
Supplement: Supplementary file 2 — Supplement 1. Technological, personnel, procedural, testing and training aspects of the tele-objective structured clinical examination. [file jeehp-18-23-suppl1.docx]

**Supplement 1. Technological, personnel, procedural, testing and training aspects of the TeleOSCE**

**Technology**

The application selected to run our online was OSCE Microsoft (MS) Teams™. The primary function utilised was the video conference meeting, but chat functions, screen sharing and recording were also required.

An online organiser (see Personnel below) created multiple separate but contemporaneous MS Teams™ meetings. These acted as virtual assessment and waiting rooms for each station. In each MS Teams™ meeting invite for each virtual assessment room were included the clinical examiner, patient (simulated or real), information technology (IT) support person and onsite organiser (see Personnel below). These digital meeting requests could also include other information for the relevant participants, such as: order of students presenting to that particular station and specific station information including any documentation to be shared during the station.

To aid communication amongst the various staff involved a group chat was also created within the MS Teams™ application. This was used to keep station timing synchronised, report and solve IT or equipment issues or raise other concerns specific to each station.

MS Teams™ allows recording of every meeting created and this was utilised as a security precaution. Students and other participants were informed that all stations would be recorded, with consent gained prior to the examination sessions commencing.

**Personnel and procedural**

Traditional in-person OSCEs require one examiner and one patient per station as well as an onsite organiser and an examination supervisor. For the teleOSCE additional personnel were required, namely: IT support, an online organiser and a station marshal table 4).

Procedurally, the in-person and teleOSCEs were largely similar with respect to the core skills assessed and the marking criteria. Due to concerns regarding timing of the overall examination the number of stations was reduced to seven in the online version, with the deletion of the extra Medicine and Surgery stations.

**Testing and training**

During development of the teleOSCE significant documentation was undertaken to capture the changes mentioned above. This was then turned into training manuals for each role and training sessions were conducted with staff involved in the examinations. Early training sessions revealed some improvements that could be made and using feedback from all parties, adjustments were made, and further testing and training was conducted. A ‘mock teleOSCE’ was then conducted for all students who wished to participate. Student and other feedback was received, problems identified and managed, and final procedural documents for the exam process were created for all participants and roles: students, examiners, patients, the online organiser, the onsite organiser and station marshal (Table 4). Only when the teleOSCE set up and procedures were established was the real summative online version undertaken.

**Table 4. Roles of personnel in the teleOSCE**

| Stage | Set up phase (days and hours ahead) | During examination | Post examination (hours) |
| --- | --- | --- | --- |
| Student | - Read relevant assessment and procedure documents  - Attend mock teleOSCE, ensuring all equipment is working, internet connection is stable and familiar with process  - Accept waiting room meeting invite | - Accept call into station  - Verbalise code of conduct and complete integrity check (first station only)  - Show clean whiteboard to station examiner  - ‘Pin’ client to screen  - Perform assessed tasks as per station requirements  - Return to waiting room at end of station  * Then repeat for each station | N/A |
| Patient | - Read relevant assessment and procedure documents  - Attend training session(s) ensuring all equipment is working, internet connection is stable and familiar with process  - Accept station meeting invite | - Respond to student as per the station scenario and student instruction | N/A |
| Examiner | - Read relevant assessment and procedure documents  - Attend training session(s) ensuring all equipment is working, internet connection is stable and familiar with process  - Accept station meeting invite  - Read information document on use of assessment form | - Welcome student to station  - Turn off camera and mute microphone, and commence student assessment  - Finalise assessment of student | N/A |
| Online Organiser | - Create MS Teams™ station and waiting room meetings  - Create separate group chat for examiners, onsite organiser and IT support  - Ensure students, patients and examiners familiar with relevant checklists and procedures  - Set up timers  - Create and open full examination schedule and group chat  - Create script for Marshals with student lists | - Start waiting room recording  - Welcome students to online waiting room and answer any last-minute questions  - Coordinate station timing, including associated messaging to station marshals  - Manage transition periods, extending them when needed (e.g. if student, client or examiner was having technical issues)  - Monitor student conduct in waiting room and any rest station  - Make record of any ‘flagged’ students or stations (e.g. for suspected integrity issues) | - Assist with removing students from any stations  - Remove student access to station recordings on Microsoft Stream |
| Onsite Organiser | - Equip stations  - Ensure all those who are to be on site are in the appropriate location  - Accept station meeting invite | - Assist with any onsite issues e.g. with equipment, patients, examiners or students | - Pack down all equipment |
| Station Marshal | - Accept station meeting invite  - Assist examiner in running the allocated station  - Ensure correct student attends in correct timeslot  - Set up timer | - Invite student into station  - Start station recording  - Check audiovisual connection is good  - Perform integrity check (first student only)  - Monitor assessor chat  - Screen-share station information document and manage station timing  - Screenshare additional resources with student on request from examiner  - Convey relevant messages to student (e.g. 2 minute warning and end of station)  - Ensure students leave station and return to waiting room in timely manner  - Finish station, check student has cleaned whiteboard and ask student to return to the waiting room  - Check examiner has completed assessment and is ready for next student | - Remove student access to station recording on Microsoft Stream |
| IT Support | - Assist sourcing IT equipment  - Troubleshoot any set up IT issues  - Accept multiple station and waiting room meeting invites | - Monitor MS Teams™ chats for anyone reporting technical issues and assist as required | N/A |
